# Amitriptyline-Induced Insomnia in a Young Lady Diagnosed With Cyclic Vomiting Syndrome: A Case Report

**DOI:** 10.7759/cureus.43249

**Published:** 2023-08-09

**Authors:** Khalid I AlHussaini

**Affiliations:** 1 Department of Internal Medicine, Imam Mohammad Ibn Saud Islamic University, Riyadh, SAU

**Keywords:** amitriptyline, saudi arabia, case report, insomnia, vomiting, cyclic vomiting syndrome

## Abstract

The author reports a 19‐year‐old woman suffering from repeated episodes of non-bloody vomiting for 18 months. All routine and special investigations were normal. She was labeled as a case of cyclic vomiting syndrome (CVS), and she developed insomnia after the initiation of amitriptyline as a prophylactic treatment. The case was reported to increase awareness regarding the importance of monitoring medication side effects among clinicians when using different classes of medications to treat CVS.

## Introduction

The author reports a 19‐year‐old woman suffering from repeated episodes of non-bloody vomiting for 18 months. All routine and special investigations were normal. She was diagnosed with cyclic vomiting syndrome (CVS) and treated with lifestyle modifications, antiemetic, and benzodiazepine as an abortive treatment, and started on amitriptyline for prophylaxis, but she developed insomnia as a side effect.

CVS is an evolving idiopathic disorder that has been reported in a limited number of countries [1]. It is quite rare among the Saudi population with a frequency of 2.6 per 1000 based on a study by Ayoola [2]. Globally, it was initially described by Heberden in 1806 in France and by Gee in 1882 in Britain [1]. CVS is an idiopathic functional disorder of gut-brain interaction (DGBI) that is characterized by recurrent attacks of nausea and vomiting combined with relatively symptom-free intervals [3,4]. It is being increasingly recognized in adults and the prevalence of CVS among the adult population was 0.7%, 1%, and 2% in Canada, the United Kingdom, and the United States, respectively, based on various population-based studies [5]. Furthermore, the incidence of CVS was reported at 3.15 per 100,000 in a survey-based study of 1,647 people in Ireland [6]. Studies showed that gender distributions varied among nations [7,8]. There are four phases of CVS, i.e., the prodromal, the emetic phase, the recovery phase, and the asymptomatic phase, as defined by Fleisher et al. [9]. However, many patients are improperly diagnosed to have gastritis, gastroesophageal reflux disease (GERD), or gastroparesis, which will lead to a delay in management [10]. A personal or familial history of migraine headaches has been labeled as a relevant associated condition in previous studies [3,4,11]. The clinical presentation consists of stereotypical bouts of vomiting with intervening periods of normal or baseline health; however, that may differ from case to case [3-5]. The patient may maintain a regular life with the help of appropriate pharmaceutical therapy and appropriate lifestyle changes [9]. Since the management of CVS relies on different pharmacological classes, it is very important to monitor patients for any unwanted adverse events. Here, the author aims to report a case of CVS in Saudi Arabia that started on amitriptyline and developed insomnia as a limiting adverse event.

## Case presentation

I had the pleasure to take the medical care of a 19-year-old female who had no history of drug abuse and suffered from multiple, discrete attacks of vomiting following her menstrual cycle and after severe stressful events, spanning roughly four to five days with symptoms-free intervals in between. The lady had previously presented to her family physician who diagnosed her with gastritis, and she was prescribed a proton-pump inhibitor and antiemetics, but these did not alleviate her symptoms. She had been complaining of recurrent attacks of non-bloody vomiting for 18 months, four to five days before the onset of menses or stress, as reported by the patient and her mother. During each episode, she would have nausea initially then develop four to five times vomiting and be unable to tolerate oral intake. Following the illness attack, she had heartburn and throat discomfort as a consequence of her repeated vomiting. These episodes were associated with intermittent nonspecific headaches and anxiety. There was no personal or family history of migraine headaches. These attacks were limiting her from her daily activity. She visited the emergency department several times for proper hydration and antiemetics. She would frequently miss her college lectures during those attacks and her symptoms have become worse over time. During her visit to the gastroenterology clinic, she had routine tests, including a complete blood count, full metabolic panel, thyroid function test, fasting blood sugar, and glycosylated hemoglobin (HbA1c), and all were unremarkable. Urine toxicology was not done, as it was not available at that time. Ultrasonography of the upper abdomen was normal, and upper gastrointestinal endoscopy came out normal with negative *Helicobacter pylori* testing. Based on her presentation, the patient was diagnosed with CVS based on adult Rome IV criteria, and the decision was to start her on amitriptyline 25 mg orally at bedtime as a preventive measure and was given an outpatient appointment in two weeks. On further follow-up, the patient was symptom-free from a CVS perspective, but she reported a development of insomnia, which she had never been concerned about before. Amitriptyline was discontinued, and a few days later, her sleep pattern improved. The patient started on levetiracetam 1000 mg orally once a day. The patient was followed up on a regular basis, and at the six-month appointment, she was well compliant with her medication, living a normal life, and free of any symptoms or pharmaceutical side effects.

## Discussion

CVS is an idiopathic illness characterized by recurrent self-limited and similar episodes of nausea and vomiting alternating with periods of baseline health (period of symptoms-free) [2-4]. It affects both children and adults [6-9]. The diagnosis of CVS is based on the historical criteria and exclusion of the alternative diagnosis [10]. A personal or family history of migraine headaches further supports the diagnosis [11]. The diagnosis of CVS is based on Rome IV criteria, first developed in 2006 and then revised later in 2016 [12,13]. Rome IV adult criteria consist of three criteria as the following: stereotypical episodes of vomiting regarding onset and duration with three or more discrete episodes in the previous year and two episodes in the past six months, occurring at least one week apart. Also, there is an absence of typical vomiting between the episodes [12,13].

CVS diagnosis might be difficult and lengthy since the symptoms are frequently encountered with many other gastrointestinal conditions and due to the lack of a particular diagnostic laboratory test or imaging. As a result, diagnosis mislabeling is possible, as seen in this case. The aim of reporting such cases is to emphasize appropriate circumstances to suspect CVS and to highlight an unusual side effect of using amitriptyline as a treatment prophylaxis. The 19-year-old college student had symptoms of multiple, separate attacks of vomiting following her menstrual period and after severe stressful events, spanning roughly four to five days with symptoms-free intervals in between. After a thorough evaluation, she was labeled as a case CVS, and the patient was counseled regarding the diagnosis, triggers, and importance of treatment compliance. During her last attack, the patient was prescribed intravenous ondansetron, intravenous omeprazole, intravenous lorazepam, and intravenous normal saline. Following the resolution of her last attack and taking into consideration her repeated episodes of CVS, the decision was made to start her on amitriptyline 25 mg oral at bedtime for prophylaxis. Three weeks later, the patient presented to the gastroenterology clinic complaining of short-term insomnia with difficulty falling asleep that she had not previously experienced. Upon interviewing the patient, she denied using any new medication apart from amitriptyline. Also, she denied any unusual stressful events during that time that could explain her sleepiness difficulties.

On reviewing the literature and to the best of the author’s knowledge, this is the first case to be diagnosed with CVS that experienced short-term insomnia with difficulty falling asleep as a side effect of amitriptyline [11]. Despite lacking randomized controlled studies, amitriptyline is generally accepted as the first-line treatment for CVS [14]. Amitriptyline is an example of a tricyclic antidepressant (TCA) drug, which blocks the normal reuptake of norepinephrine and serotonin neurotransmitters. Amitriptyline is a tertiary amine with robust binding affinities for alpha-adrenergic, muscarinic (M1), and histamine (H1) receptors [14]. The use of amitriptyline in adults with CVS was linked to a reduction in emergency department visits and hospitalizations [15]. Amitriptyline is well-known and approved for a long time as an effective medication to treat depression in adults. Furthermore, there are few non-approved trials to treat anxiety, post-traumatic stress disorder, fibromyalgia, irritable bowel syndrome, and postherpetic neuralgia. The common side effect of amitriptyline includes orthostatic hypotension, tachycardia, arrhythmia, dry mouth, urinary retention, glaucoma, weight gain, and abnormality in liver enzymes [14].

Amitriptyline had been discontinued, and the patient was followed two weeks later, and she reported an improvement in her sleep pattern beginning one week after the medication withdrawal. The patient was started on levetiracetam 1000 mg orally once a day. The patient was followed up on a regular basis, and at the six-month appointment, she was well compliant with her medication, living a normal life, and free of any symptoms or pharmaceutical side effects.

## Conclusions

As far as the author is aware, this is the first reported case of CVS that developed insomnia as a side effect after amitriptyline use in Saudi Arabia, which would create awareness regarding the importance to monitor medication side effects among clinicians when using different classes of medications to treat CVS.
